# The Treatment of Neglected Perineal Lacerations*Read before the Central Kentucky Medical Society, January 20th, 1898.

**Published:** 1898-03

**Authors:** Louis Frank

**Affiliations:** Louisville, Kentucky; Visiting Gynecologist to the Louisville City Hospital; No. 129 West Chestnut Street


### Southern Medical Record.

A MONTHLY JOURNAL OF MEDICINE AND SURGERY.

VOL. XXVIII. ATLANTA, GA., MARCH, 1898. NO. 3.

### Original Articles.

### THE TREATMENT OF NEGLECTED PERINEAL LACERATIONS.*

By LOUIS FRANK, M.D., Louisville, Kentucky.

Visiting Gynecologist to the Louisville City Hospital

Very few women who bear children escape without lacerations of the perineum; consequently we find that more married women come to the gynecologist suffering from lacerations of the perineum than from any other pathological condition.

The subject has been very much overlooked of late, not only by writers, but also by operators, probably because they have sought the name and reputation which are more easily to be derived from major surgery. I know of no one subject of more interest which the general practitioner and specialist might discuss, as here they are upon neutral ground, as it were, one upon which they can touch hands. I have heard of men who have gone through an active obstetrical practice extending over a number of years without ever having seen a lacerated perineum among their own clientele. I grant you that a woman may occasionally give birth to a child without a rupture, but when men make such statements as this to intelligent doctors, we must come to the conclusion that either they have delivered no women, have never examined the perineum after labor to see whether or not a laceration has occurred, or they have not been honest enough to admit to themselves the actual condition. It is in just such conditions that an honest and careful accoucher can, by an acknowledgment which does him no harm, and by immediate treatment prop-

*Read before the Central Kentucky Medical Society, January 20th, 1898.

erly applied, often save his patient hours of suffering and the confinement and other objectionable features of a subsequent operation. "Murder will out" and conditions arise as a result of certain kinds of tears which will eventually compel the patient to seek relief.

As to why some births should be accompanied by extensive laceration, others with slight ones, of one variety or the other, I will not discuss, but will confine my remarks rather to the consequences thereof, and the treatment therefor.

The subsequent condition of the genital tract, and I might say of the generative organs, depends upon the character or direction of the perineal tear. A glance at the anatomy of the pelvic floor will make clear the reasons. I will first say that tears may occur in the median line, extending not deeply into the vagina, but involving the external structures, or they may extend in the median line some distance into the vagina, at times going through even into the rectum. In other cases we find that the tear has begun in one or both vaginal sulci extending towards the osteum vaginæ, and in a direction converging towards the median line, thence through the skin and possibly the anal muscle to the rectum. We therefore divide lacerations of the perineum into median and lateral. We may make a further division according to the extent of the tear into incomplete and complete lacerations.

I would call an incomplete tear one which extends to the sphincter muscle, but not involving any of its fibres. A complete tear extends through the sphincter muscle, and may or may not enter the bowel. There may be slight lacerations involving only the fourchette. We also have tears known as transverse, which most often, though not always, occur without any rupture of the mucous membrane. Tears occurring exactly in the median line may never give rise to any serious consequences. The woman may never consult a physician for these lacerations, even though extensive, provided they do not involve the sphincter muscle, and are unaccompanied by incontinence of feces. Neglected lacerations involving either or both of the lateral sulci, extensive or slight, give rise sooner or later to very unpleasant consequences, such as may make the woman very uncomfortable, or even a confirmed invalid, and if a doctor is not consulted it is purely from modesty or because the woman thinks these things are natural to all mothers.

To understand the effect of a laceration it is essential that we should bear in mind the anatomy of the perineum. We find going from the external or skin surface that we come to two layers of fascia, a superficial and a deep layer. Under this we find extending from each lateral pelvic wall, arising from the bony structures and attached to the lateral vaginal wall, two muscles, viz., the transversus perinei. Extending around in an anteposterior direction, arising from tendinous tissues about the clitoris, we find the sphincter vaginæ, or bulbo-cavernosi muscles, under these the anterior and posterior layer of the triangular ligament, and last and probably more important than any, the levator ani. The fibres of the transversus perinei coalesce, to some extent, with those of the sphincter vaginæ, and acting in unison, tend to draw the vagina upward and forward; they are assisted therein by the levator ani, probably the most important of all the structures which go to make up the pelvic floor. The levator ani, arising as it does, in front, from the pubic bone, and at the sides from the tendinous arch of the pelvic fascia, passes back and encloses by its inner fibres the vagina as in a sling. Its action is similar to that of the other two muscles spoken of. It and the sphincter vaginæ are inserted in a tendinous median raphe; consequently, if a tear is directly in the median line, their fibres are not torn, and their insertion is not materially interfered with, therefore each muscle acting from its point of origin still tends to draw the vagina forward and upward, in this way supporting the pelvic structures.

If we introduce the finger within the vagina we can feel in the posterior sulci the pelvic structures which support the floor of the pelvis forming the perineum; they are felt as a tense, resisting band, about half an inch in thickness. So much for the anatomy of the perineum.

As I have said, it is the lateral tears which lacerate the fibres themselves, composing the muscular structures here, which are followed by the conditions which may cause chronic invalidism. These tears, if not repaired at once, may cause sub-involution of the vagina and the uterus, rectocele, cystocele, or even uterine prolapse, with complete descent in aggravated cases. Picture the condition!

Now what can be done to prevent or overcome this? In a certain number of cases it is absolutely impossible to avoid damage to the pelvic floor during childbirth, no matter how expert the obstetrician may be, and I take it that no man need be ashamed to acknowledge to the patient or to the world that this accident has occurred, and an honest man will acknowledge it. Early recognition of this accident is then most desirable, as much can be done at this time by immediate operation towards the prevention of any future trouble. Of course at times we will meet with failures following immediate perineorraphy, and our cases could then be subjected to intermediate, or better still, a secondary operation. Still, a failure should not deter us from operating and thus benefiting and curing many others. Should union fail to take place, or should the laceration escape notice, and the woman come later for treatment, we will find the vagina gaping, bulging of the posterior wall in laterial tears, with possibly other conditions, and cicatricial tissue occupying the site of the old rupture. By intelligent operation these women can most all be cured, no matter how severe or extensive the tear.

Two operations bid for favor, the Tait or flap splitting, and the modified Emmet. The former is, in my opinion, of little value except for cosmetic effect, though it may do in some instances of median tear. Its mechanism, however, is not correct for those involving the integrity of the muscular structure itself. The operation par excellence for these is the Emmet, and while you are all familiar with the operation, there are some points I desire to call especially to your attention.

First is the preparation of the patient: Because we do not enter the abdominal cavity, there are some who think that there is not such absolute necessity for the rigid aseptic precautions which are ordinarily taken. But disabuse your minds; it is just as essential that the vagina, what remains of the perineum, and the rectum, be just as carefully prepared as if we were about to do an hysterectomy or any other major operation. We must avoid, above all else, suppuration, for the least infection of a single suture may destroy the effect of the operation entirely.

As to the methods of preparing patients, instruments and the sutures, I shall have nothing to say, as these are too well known to require repetition. Not only, however, is local preparation necessary, but the patient should have her general system prepared, by purgation accustoming her to remain in bed, and if there is the least falling ing below par, she must be toned up before anything is done. I recently met with a failure in one of my perineorraphies on account of the low vitality of the patient generally, which I think would have been avoided had I kept her upon tonics and built her up as should have been done. So much for the preliminaries.

Now as to the operation itself: Very few instruments are needed to do a perineorraphy, no matter what the extent of the injury. Primarily, a good anterior retractor, one pair of bullet forceps, two tenacula with full curve, a mouse-tooth tissue forceps, four or five hemostatics, one pair of scissors curved on the flat, preferably pointed, a sharp knife, needle-holder and four fully curved needles, the Kelly being preferable, with silkworm gut, and catgut is all that is required. Proper denudation is of as much importance as the suturing itself. At times we must carefully examine to find the cicatrix. It can always be recognized if we will remember that in cicatricial tissue there are no rugæ as ordinarily found in the vagina. Having determined the extent of the tear, we catch the crest of the rectocele, if a rectocele exist, and one will be found in all tears involving one or both sulci, transfix the labiæ minoræ at their posterior extremity just at the orifice of the vulvo-vaginal glands, and then with our knife mark out a triangular area in each sulcus which is to be removed. The knife is then swept around at the juncture of the muco-cutaneous surface uniting the two most external points that are to be denuded. This will give us an area marked out like the letter "M" with the two perpendiculars of the M diverging and united by a curved line.

The area thus marked out is rapidly denuded, using for this purpose the mouse-tooth tissue forceps to hold the flaps, and doing the cutting with our scissors. The two sulci can in this way be easily freed of all cicatricial tissue. We will have the posterior vaginal wall left as a tongue, and this tongue should easily meet the most external angles of denudation. Hemorrhage should be carefully controlled. This having been done we are now ready to pass our sutures, which we do in the following manner:

First a silkworm gut suture, which begins on the vaginal mucous membrane, and passes down parallel to the freshened muco-cutaneous juncture, the needle emerging at the bottom of the sulcus; it is then re-entered on the other side at the bottom of the same sulcus, and passes up coming out upon the mucous surface of the tongue at a distance from its point equal to the distance which the external angle of denudation is from the point at which the suture entered. A suture is passed upon the opposite side in the same manner. These two sutures are of silkworm gut. They should be passed deep out, so as to catch the torn fascia and muscle. If they are passed just under the freshened surface they will do little or no good, but, as I say, we must go wide of the raw surface thus including the planes of fascia and the levator ani. This is very essential. These two silkworm gut sutures are now tied. They are the only sutures which are within the vagina to require removal. We will find that the calibre of the vagina is immediately lessened. The angles above these sutures are now closed in with interrupted sutures of catgut. For this purpose I use a catgut which I know to be sterile, and one upon which I have been depending for some time for abdominal work, which requires no further preparation and can be taken and used directly from the vials in which it comes. These sutures are made by the J. Ellwood Lee Company. For this work I use a No. C.

The next suture introduced is what is known as the Crown suture, and it is the one which lifts up the perineal floor to the level of the vagina and restoring it as nearly as it is possible to do. These sutures begin on the skin surface, and are passed in below the external angles. They are passed outwards and upwards, emerging just under the mucous membrane of the vagina on the denuded surface. They cut the external angle, as it were. This suture is now passed under the mucous membrane of the tongue directly through it, transfixing it, then enters the denudation upon the opposite side just under the mucous membrane, and outward again upon the skin at a point corresponding to the one where it was first passed in. This is the third and last silkworm-gut suture which we use. We will now have an open space beneath this crown suture, which is closed in with cat-gut, sweeping the needle well out and passing it through the tongue each time. Two or three sutures will be all that are necessary. The tip of the tongue can be now brought in a position with the tip of the denuded angle with catgut sutures.

Those of you who have never seen the operation performed in this way will find it a revelation. It is almost impossible to conceive of the difference which occurs in the appearance of the vaginal outlet when this crown suture is tied. At times it seems as if you can not get your anterior retractor out. Examination digitally will reveal a thick, strong and firm perineum.

The retractor being withdrawn, the vagina is dried with a small sponge, the suture lines are dusted with iodoform or any other dressing powder that may be preferred, and the patient put to bed. Douches are not necessary, all that is required is to keep the lines of union as dry as possible. You are not annoyed, nor will your patient be, by the removal of vaginal sutures, as there are only two intra-vaginal to be removed, and these are near the orifice; the other suture to be removed is external.

The after care of the patient consists in catheterization every six or eight hours for the first few days; after that seeing that no urine escapes upon the line of union, and to move the bowels on the third or fourth day. This is best done by the administration of salines in small doses every hour, and the administration of an anema of warm water to soften the fecal matter as soon as the patient feels a desire to defecate. The sutures can be removed on the tenth day. I make it a rule to keep the patient in bed for two and a half to three weeks.

The point in the operation to which I would especially call your attention, is the method of suturing, it differing from that originally devised by Emmet. In all operations heretofore, there has been no fixed point, hence no "lifting up." The sutures introduced in the manner described have the lateral walls as a point towards which traction is made, and thus overcome this heretofore mechanical defect of the operation. It is for just this reason that the old operation gave only cosmetic effects, unless the perineum has been built up layer by layer from below as has been and is still done by some operators, uniting the structures separately.

The repair of median lacerations is so simple and easy that little will be said upon that subject. I would only state that all scar tissue must be gotten rid of, and that if the laceration extends any distance up the vagina, be sure to unite the mucous surfaces and avoid all pocketing; pass the sutures well out and well under the raw areas.

If the sphincter muscle is involved, its retracted ends can be located by feeling upon each side a pit-like depression. You will find that the corrugation, which is normal about the anus, will have disappeared except over an area corresponding to the area of the retracted muscle. The ends of the muscle must be denuded by picking up the tissue with forceps and snipping it out. The sutures are then passed upon the skin side well out, going through the muscle up under the mucous membrane to the border of the rectal mucosa, and out at a corresponding point on the other side. When these two sutures are tied, the rectum may seem to be drawn backward; a suture may even disappear within the anal orifice. We may know that the muscle has been caught and the torn ends approximated by recognizing a restoration of the anal corrugation.

If the mucous membrane of the bowel is torn through, it must first be united with buried catgut sutures before the perineum is restored, and it is well in cases of this sort not to allow the bowels to move until about the fifth day.

There is just one condition that sometimes follows laceration, of which I desire to say a few words, i. e. prolapsus uteri. If the prolapsus is slight, and the organ has not descended much, repair of the perineum in this manner will often answer every purpose and effect a permanent cure; after the perineum is repaired, ventro suspension should be done. If the cervix is elongated, it should be amputated. If the prolapsus is complete, the only thing, in my opinion, that can be done is an hysterectomy. Ventro fixation, ventro suspension, nor any other operation will retain these uteri in their normal position; prolapsus will recur sooner or later and the woman will continue to lead a miserable existence until the organ is completely removed.

*No. 129 West Chestnut Street.*

